# Retrospective Evaluation of the Prognostic Utility of Clinical and Laboratory Findings in Hospitalized Cats with Pancreatitis

**DOI:** 10.3390/ani15071060

**Published:** 2025-04-06

**Authors:** Yada Siriphanporn, Anuwat Wiratsudakul, Suwicha Kasemsuwan, Piyathip Chuchalermporn, Monchanok Vijarnsorn, Narudee Kashemsant

**Affiliations:** 1 Internal Medicine Unit, Kasetsart University Veterinary Teaching Hospital, Faculty of Veterinary Medicine, Kasetsart University, 50 Ngamwongwan Rd., Lat Yao, Jatujak, Bangkok 10900, Thailand; yada.siri@ku.th; 2 Department of Clinical Sciences and Public Health, Faculty of Veterinary Science, Mahidol University, 999 Phutthamonthon Sai 4 Road, Salaya, Phutthamonthon, Nakhonpathom 73170, Thailand; anuwat.wir@mahidol.ac.th; 3 Department of Veterinary Public Health, Faculty of Veterinary Medicine, Kasetsart University, Kamphaeng Saen Campus, Nakhon Pathom 73140, Thailand; fvetswk@ku.ac.th; 4 Radiology Unit, Kasetsart University Veterinary Teaching Hospital, Faculty of Veterinary Medicine, Kasetsart University, 50 Ngamwongwan Rd., Lat Yao, Jatujak, Bangkok 10900, Thailand; piyathip.c@ku.th; 5 Department of Companion Animal Clinical Sciences, Faculty of Veterinary Medicine, Kasetsart University, 50 Ngamwongwan Rd., Lat Yao, Jatujak, Bangkok 10900, Thailand; fvetmov@ku.ac.th; 6 Department of Physiology, Faculty of Veterinary Medicine, Kasetsart University, 50 Ngamwongwan Rd., Lat Yao, Jatujak, Bangkok 10900, Thailand

**Keywords:** cats, clinical epidemiology, pancreatitis, prognosis, retrospective study

## Abstract

Pancreatitis in cats ranges from mild forms with low mortality to severe cases with poor prognosis, and early identification of high-risk patients remains a challenge. The prognostic significance of hyperlactatemia and metabolic acidosis in feline pancreatitis is not well established. This study aimed to identify prognostic factors in hospitalized cats with pancreatitis and assess the predictive value of hyperlactatemia and metabolic acidosis. A retrospective study of 142 cats diagnosed between January 2020 and December 2023 was conducted, analyzing signalment, clinical signs, and clinicopathological findings. Diagnosis was based on clinical signs, abnormal feline pancreatic lipase assay, and abdominal sonography. Multivariate analysis identified jaundice (OR, 4.6; 95% CI, 1.7–12.7), renal disease (OR, 2.0; 95% CI, 1.05–5.40), hypoalbuminemia (OR, 3.91; 95% CI, 1.70–9.01), increased blood urea nitrogen (BUN) (OR, 4.76; 95% CI, 1.96–11.53), and neutrophil cytoplasmic toxic change (OR, 10.54; 95% CI, 4.17–26.7) as independent prognostic indicators. The combination of renal disease and jaundice increased the mortality risk 12-fold, while hypoalbuminemia and neutrophil toxic changes increased it 120-fold. However, hyperlactatemia and metabolic acidosis upon admission were not predictive of outcomes. These findings suggest that jaundice, renal disease, hypoalbuminemia, increased BUN, and neutrophil toxic changes are significant prognostic indicators in feline pancreatitis, whereas hyperlactatemia and metabolic acidosis do not predict mortality.

## Simple Summary

Pancreatitis is the most common exocrine pancreatic disease in cats. Since most feline pancreatitis cases are idiopathic, treatment is primarily supportive and symptomatic. Rapid identification of high-risk patients allows for timely decisions to implement aggressive medical and nutritional interventions at an early stage. This study used the medical records of 142 cats diagnosed with pancreatitis to identify potential markers of disease prognosis. The study aimed to evaluate the potential prognostic significance of clinical signs, physical examination findings, and clinicopathological data, focusing on the roles of metabolic acidosis and lactic acidosis as prognostic factors in feline pancreatitis. We found that jaundice, concurrent renal disease, hypoalbuminemia, increased blood urea nitrogen, and neutrophil cytoplasmic toxic change at the time of hospital admission were independent prognostic indicators of the outcomes.

## 1. Introduction

Pancreatitis is the most common exocrine pancreatic disease in both dogs and cats [1]. The reported mortality rate of feline pancreatitis ranges from 9% to 41% based on data from four studies [2,3,4,5]. As a severe catabolic condition, pancreatitis can lead to systemic inflammatory response syndrome (SIRS), resulting in multiple organ dysfunction and, potentially, death [6]. In cats, the prognosis of pancreatitis is closely associated with disease severity and duration, the extent of pancreatic necrosis, the development of local and systemic complications, and the presence of concurrent diseases [7].

Several scoring systems and biomarkers have been investigated in both human and veterinary medicine to predict the prognosis of pancreatitis. These aim to identify high-risk patients early, allowing clinicians to implement aggressive medical and nutritional interventions at an earlier stage [7,8,9]. However, no single prognostic factor has been definitively established. Retrospective studies in feline populations have identified low-plasma ionized calcium concentrations, hypoglycemia, and azotemia as negative prognostic indicators in cats with acute pancreatitis (AP) [4,5,7,10]. Metabolic acidosis (MA) has been recognized as a prognostic factor for AP [11,12,13].

A 2018 retrospective study found that feline non-survivors of pancreatitis frequently exhibited low serum total CO_2_ (TCO_2_) concentrations, suggesting a strong association between acidemia and metabolic acidosis [4]. These findings indicate that systemic pH imbalances may play a significant role in the progression of AP. However, the mechanisms linking AP to systemic pH disturbances remain incompletely understood. Proposed mechanisms include direct causes, such as the loss of bicarbonate-rich pancreatic secretions through fistulas or drainage, and indirect causes, such as lactic acidosis associated with shock, sepsis, cardiovascular failure, or gastrointestinal bleeding [14].

To our knowledge, the prognostic impact of hyperlactatemia and metabolic acidosis in feline pancreatitis has not been previously investigated. A comprehensive evaluation of systemic complications and laboratory abnormalities—particularly blood gas analysis to detect metabolic acidosis and hyperlactatemia—may provide valuable insights into improving treatment strategies and prognosis. Therefore, this study aimed to assess the prognostic significance of clinical signs, physical examination findings, and clinicopathological data, with a particular focus on the roles of metabolic acidosis and lactic acidosis in feline pancreatitis.

## 2. Materials and Methods

### 2.1. Study Population and Inclusion Criteria

This retrospective study included medical records of client-owned cats diagnosed with acute pancreatitis at the Kasetsart University Veterinary Teaching Hospital in Bangkok, Thailand. The database was reviewed for cases presented between January 2020 and December 2023. Cats were eligible for enrollment if they met the following inclusion criteria: a history of clinical signs such as lethargy, anorexia, vomiting, diarrhea, and/or weight loss; physical examination findings consistent with pancreatitis, including dehydration, hypo- or hyperthermia, icterus, and/or abdominal pain, as defined by the ACVIM consensus (2021); a positive SNAP fPL test (Feline SNAP fPLI, IDEXX Europe B.V., Hoofddorp, The Netherlands); the presence of at least two sonographic abnormalities, as reviewed by a board-certified radiologist, which includes pancreatomegaly, alterations in pancreatic echogenicity and echotexture, irregular pancreatic contours, a hyperechoic mesentery, peripancreatic fluid accumulation, or irregular or abnormal pancreatic duct dilation.

### 2.2. Data Collection and Clinical Assessments

On the day of presentation, data recorded included signalment, clinical signs, physical examination findings, concurrent diseases, and clinicopathological data. The complete blood count (CBC) was analyzed using the Sysmex XN-1000^®^ (Sysmex Corp., Kobe, Japan). Serum biochemistry parameters, including blood urea nitrogen (BUN), serum creatinine (Scr), alanine aminotransferase (ALT), alkaline phosphatase (ALP), total protein (TP), albumin (Alb), and total bilirubin, were measured using the ILab Tarsus analyzer (Instrumentation Laboratory, Milan, Italy). Venous blood gas and lactate analyses were performed using the Techno Medica GASTAT 700 series (Kanagawa, Japan).

In this study, concurrent disease was defined as the presence of two or more coexisting medical conditions. Gastrointestinal (GI) and GI-associated diseases included disorders affecting the liver, pancreas, or intestines. Renal disease was defined by the presence of azotemia and abnormal renal parenchyma upon ultrasonographic examination.

Survivors were defined as cats that were discharged alive, whereas non-survivors included those that either died or were euthanized during hospitalization due to clinical deterioration despite ongoing treatment. Cats whose owners opted to discontinue treatment were also classified as non-survivors.

### 2.3. Statistical Analysis

All statistical analyses were performed using commercial software (NCSS 11, NCSS LLC., Kaysville, UT, USA). The Kolmogorov–Smirnov test was used to assess the distribution of quantitative variables. Normally distributed variables are presented as mean ± standard deviation (SD), while non-normally distributed variables are expressed as median and interquartile range (IQR). Comparisons between two independent groups were conducted using the Student’s *t*-test for normally distributed variables and the Mann–Whitney U test for non-normally distributed variables. Associations between qualitative variables were evaluated using the χ^2^ test or Fisher’s exact test. A *p*-value < 0.05 was considered statistically significant.

Univariate logistic regression analysis was initially performed to identify potential risk factors associated with mortality. Significant factors (*p* < 0.2) were then incorporated into a multiple logistic regression model using backward selection to generate a predictive equation for mortality. The significance level for the final model was set at *p* < 0.05. The first set of models assessed clinical signs and physical examination findings as predictors of mortality. A second set of models evaluated clinicopathological data obtained on the day of presentation, including CBC, blood chemistry, and blood gas analysis, using multivariable logistic regression analysis.

## 3. Results

A total of 142 client-owned cats were included in this study, comprising 65 males (45.7%) and 77 females (54.3%). The majority were domestic shorthair (100; 70.4%), followed by Persian (26; 18.3%), Scottish Fold (7; 4.93%), and other breeds (7; ≤2 cats each). The mean age was 9.3 ± 4.6 years. There were no significant differences in breed (*p* = 0.1), age (*p* = 0.17), or sex (*p* = 0.37) between survivors and non-survivors. However, the mean body weight (BW) was significantly higher in survivors than in non-survivors (4.5 ± 1.5 kg vs. 3.8 ± 1.4 kg, *p* = 0.0004).

Of the 142 cats, 63 (44.3%) survived, while 79 (55.7%) did not. Among the non-survivors, 68 out of 79 (86%) died during hospitalization, while 11 of 79 (13.9%) were euthanized due to clinical deterioration or owner-directed withdrawal of treatment. Concurrent diseases were documented in 135 of 142 cats (95.1%) (Table 1). The most common concurrent conditions were gastrointestinal (GI) and GI-associated diseases (67/142; 47.2%), including cholangiohepatitis (28/67), enteropathy (21/67), and triaditis (16/67). Other common conditions included renal disease (32.4%), endocrinopathy (27.5%), cardiac abnormalities (12.7%), and post-operative care (12.0%). Post-operative care was more frequently identified in survivors than in non-survivors (*p* = 0.02).

The most frequently observed clinical signs were lethargy (122/142; 85.9%), anorexia (117/142; 82.4%), and vomiting (64/142; 45.1%). Other signs included diarrhea and weight loss. Common physical examination findings included dehydration (115/142; 81.0%), hypothermia (110/142; 77.5%), and abdominal pain (100/142; 70.4%), while icterus and fever were less frequent. No significant differences in clinical signs were found between survivors and non-survivors. However, icterus was significantly more common in non-survivors (*p* = 0.009) (Table 2).

Complete blood count (CBC) abnormalities included anemia (91/142; 64.1%), thrombocytopenia (83/142; 58.4%), and leukocytosis (66/142; 46.5%) (Table 3). Neutrophil cytoplasmic toxicity was observed in 61 cats (42.9%). Significant differences were noted in hematocrit and red blood cell count (RBC) between survivors and non-survivors (*p* = 0.014 and *p* = 0.043, respectively). Neutrophil cytoplasmic toxic changes were significantly more frequent in non-survivors (50/79; 63.3%) than in survivors (11/63; 17.5%) (*p* < 0.0001).

Serum biochemistry abnormalities were observed in most cats, including hyperbilirubinemia (41/44; 93.2%), elevated aspartate transaminase (AST) (18/19; 94.7%), elevated alkaline phosphatase (ALP) (82/136; 60.3%), hyperphosphatemia (91/132; 68.9%), hyperbilirubinemia (80/142; 56.3%), and hypoalbuminemia (70/142; 49.3%) (Table 3). Blood urea nitrogen (BUN) levels > 34 mg/dL were more frequent in non-survivors (47/79; 59.5%) than in survivors (21/63; 33.3%) (*p* = 0.002). Similarly, elevated serum creatinine (>1.6 mg/dL) was more common in non-survivors (45/79; 57%) than in survivors (24/63; 38.1%) (*p* = 0.0254). Hypoalbuminemia (<2.8 g/dL) was significantly more frequent in non-survivors (51/79; 64.6%) than in survivors (19/63; 30.2%) (*p* < 0.001). Significant differences were also observed for BUN, creatinine, total protein, and albumin between survival groups (*p* = 0.003, *p* = 0.044, *p* = 0.01449, and *p* = 0.00003, respectively) (Table 3).

Venous blood gas analysis on the first day of hospitalization revealed abnormalities, including low bicarbonate (hypoHCO_3_) (39/103; 37.9%), hyperlactatemia (34/103; 33.0%), hypocalcemia (29/103; 28.2%), hypokalemia (24/103; 23.3%), and acidemia (24/103; 23.3%) (Table 4). Blood pH and bicarbonate (HCO_3_) levels were significantly different between survivors and non-survivors (*p* = 0.0123 and *p* = 0.0280, respectively). The frequency of acidemia (pH < 7.22 mmol/L) was higher in non-survivors (19/60; 31.7%) than in survivors (5/46; 11.6%) (*p* = 0.0177). Similarly, hypoHCO_3_ (<18 mmol/L) was more common in non-survivors (29/60; 48.3%) than in survivors (10/43; 23.2%) (*p* = 0.0097).

A total of 28 variables recorded at presentation were initially analyzed for their association with mortality using a univariable logistic regression model. Variables with *p* < 0.2 were subsequently included in backward multivariable analysis. In the model based on clinical signs and physical examination findings, only icterus (odds ratio [OR], 4.6; 95% confidence interval [CI], 1.7–12.7; *p* = 0.002) and concurrent renal disease (OR, 2.0; 95% CI, 1.05–5.40; *p* = 0.038) remained significantly associated with increased odds of mortality. In the model based on clinicopathological data, only hypoalbuminemia (OR, 3.91; 95% CI, 1.70–9.01; *p* = 0.046), increased BUN (OR, 4.76; 95% CI, 1.96–11.53; *p* = 0.003), and neutrophil cytoplasmic toxic change (OR, 10.54; 95% CI, 4.17–26.7; *p* < 0.0001) remained significant predictors of mortality (Table 5). No significant correlations were found among variables in the final model.

## 4. Discussion

Cats with pancreatitis showed no age, sex, or breed predisposition, consistent with the ACVIM consensus published in 2021 [10]. The mean body weight at presentation was lower among non-survivors. However, the mean body weight in both groups remained within the healthy range previously reported for Thai domestic shorthairs, where the median body weight was 3.95 kg (range: 2.2–6.0 kg) for males and 3.30 kg (range: 1.7–5.8 kg) for females [15]. This weight difference may suggest greater weight loss among non-survivors. However, as body condition score (BCS) and muscle condition score (MCS) were not assessed in this study, future research should incorporate these evaluations to better understand the nutritional status of affected cats.

Concurrent diseases were identified among most cats in this study, consistent with previous reports in which pancreatitis in cats was frequently associated with hepatobiliary disease, triaditis, and renal disease [16,17,18,19]. Cats with concurrent diseases were not excluded, as it is a common finding in feline pancreatitis and was one of the factors evaluated as a potential prognostic indicator. Icterus at presentation was more frequently observed in non-survivors. This finding is plausible, given that cholangiohepatitis and triaditis were among the most common concurrent diseases. A previous study reported that cholangitis was commonly accompanied by pancreatitis (60%) and inflammatory bowel disease (50%), with 32% of cases exhibiting both conditions [20]. Similarly, triaditis has been diagnosed in 50–56% of cats with pancreatitis [21,22]. Jaundice is a common clinical manifestation that overlaps among cholangiohepatitis, pancreatitis, and triaditis in cats [22], and has also been reported in humans with gallstone pancreatitis [23]. Feline cholangitis–cholangiohepatitis syndrome (CCHS) is a necroinflammatory liver disorder that predominantly affects the portal tract. These conditions are classified as cholangitis or as cholangiohepatitis when inflammatory infiltrates extend beyond the limiting plate of the portal tract. Center et al. [24] reported that CCHS with pancreatitis is frequently associated with extrahepatic biliary duct obstruction (EHBDO), cholelithiasis, and microcholelithiasis, conditions that promote the retrograde ascension of bile and bile-borne bacteria into the common bile and pancreatic ducts, facilitate enteric bacterial translocation and systemic dissemination, and can lead to a cascade of events—including acute systemic bacteremia, endotoxemia, circulatory collapse, refractory hypotension, and progression to acute renal failure—with potentially fatal outcomes. Furthermore, findings from a 2022 study demonstrated that cats with CCHS and hyperbilirubinemia had significantly shorter long-term survival compared to normobilirubinemic cats. This highlights the potential prognostic significance of jaundice in affected feline patients and further supports the observations presented in this study. In our study, EHBDO was identified in 8 out of 67 cats (3 in the survival group and 5 in the non-survival group) based on abdominal ultrasonographic findings, including diffuse heterogeneous hepatic parenchymal changes, distension of the common bile duct and gallbladder, and increased bile sediment [19]. Similarly, cholelithiasis was diagnosed in 4 out of 67 cats (2 in the survival group and 2 in the non-survival group), with ultrasonographic evidence of echogenic material within the common bile duct, hepatic ducts, or gallbladder [24]. However, in the present study, neither EHBDO nor cholelithiasis was found to be significantly associated with survival outcomes.

Additionally, systemic inflammatory responses in pancreatitis, driven by upregulation of various proinflammatory mediators and chemokines [6], contribute to the development of multiple organ dysfunction syndrome (MODS). In human patients, even in the absence of MODS, liver injury severity has been positively correlated with acute pancreatitis severity due to factors such as liver perfusion abnormalities, NF-κB signaling pathways, and endotoxemia from intestinal dysfunction [25]. In contrast, in this study, serum bilirubin levels and the frequency of hyperbilirubinemia did not significantly differ between survivors and non-survivors. Jaundice typically manifests when bilirubin levels exceed 2.5 mg/dL [6], while the median bilirubin level in the survival group was 2.4 mg/dL. Thus, some surviving cats exhibited hyperbilirubinemia without clinical jaundice. Moreover, variability in bilirubin levels between groups may have obscured significant differences.

Concurrent renal disease was identified as a potential prognostic factor in feline pancreatitis. However, this study could not differentiate between acute kidney injury (AKI) and chronic kidney disease (CKD), as classification was based on the presence of azotemia and ultrasonographic abnormalities in the renal parenchyma. CKD is typically characterized by reduced kidney size, irregular margins, hyperechoic cortices, and poor corticomedullary differentiation, although some of these features can also be seen in aging or AKI-affected cats [26,27]. The lack of serial monitoring data, urine-specific gravity, and urinalysis results limited the ability to distinguish between AKI and CKD. The association between renal disease and poor prognosis was further supported by the higher prevalence of azotemia in non-survivors. This azotemia may have resulted from dehydration-induced reductions in renal perfusion and/or intrinsic renal disease, encompassing both AKI and CKD. In humans, dogs, and cats, pancreatitis and AKI frequently coexist, each exacerbating the other’s progression [4,9,28]. Additionally, a prior study reported an increased prevalence of CKD in cats with pancreatitis [29]. Azotemia has consistently been identified as a negative prognostic marker in multiple studies on acute pancreatitis in both dogs and cats [4,9,30,31]. Future prospective studies are warranted to determine whether CKD serves as a definitive prognostic factor in feline pancreatitis.

BUN is commonly used as a prognostic indicator in pancreatitis for both humans and dogs [32,33,34,35], although reports in cats remain limited [36]. The association between BUN elevation and increased mortality risk may be attributed to AKI, renal hypovolemia due to increased vascular permeability, and interstitial extravasation caused by inflammation associated with SIRS. Additionally, chemical injury to the kidneys, mediated by activated enzymes, inflammatory cytokines, and systemic mediators, may contribute to these renal changes [34].

Neutrophil cytoplasmic toxic change was a common hematologic abnormality in cats with acute pancreatitis [6,37] and in hospitalized cats in general [37,38,39]. Toxic neutrophils on blood smears indicate leukocyte abnormalities suggestive of systemic inflammation [38,40]. In this study, toxic neutrophil changes, rather than neutrophilia or leukocytosis, showed a significant association with mortality. This may be due to cytoplasmic toxicity serving as an early marker of disease severity, preceding leukogram abnormalities or left shifts [37,40,41]. The prognostic value of toxic neutrophil changes remains controversial. Previous feline studies found no significant difference in mortality between cats with toxic neutrophils but reported that hospitalization duration and treatment costs were significantly higher in affected cats [37,39]. These findings suggest that toxic neutrophil changes may indicate more severe systemic diseases [39]. Conversely, a study in dogs found that increasing neutrophil toxicity correlated with higher case fatality rates [40].

Hypoalbuminemia is a well-recognized clinicopathological abnormality in human acute pancreatitis [42,43,44] but is less frequently reported in canine and feline cases [5,6,7]. This study identified hypoalbuminemia as a common finding and a potential prognostic marker in feline pancreatitis. Several mechanisms contribute to hypoalbuminemia in acute pancreatitis, including (i) an energy deficit due to fasting and increased tissue catabolism, (ii) inflammatory cytokine-mediated suppression of hepatic albumin synthesis, and (iii) cytokine-induced endothelial dysfunction, leading to increased capillary permeability and albumin leakage into the interstitial space [43]. In humans, the C-reactive protein-to-albumin (CRP/ALB) ratio has been investigated as a prognostic marker, with higher ratios at presentation correlating with increased mortality [45]. Similarly, in dogs, elevated CRP/ALB ratios have been associated with increased mortality risk in acute pancreatitis [46]. However, CRP has been deemed an unreliable biomarker in feline medicine [47,48]. Future studies should focus on identifying feline-specific acute-phase proteins and their relationship with albumin as potential prognostic markers in feline pancreatitis.

The final model identified several independent risk factors for predicting mortality in feline pancreatitis. In the model based on clinical signs and physical examination findings, the presence of concurrent renal disease and jaundice was significantly associated with increased odds of mortality. Cats presenting both conditions had a 12-fold higher risk of death compared to those without renal disease and jaundice. In the model based on clinicopathological data, hypoalbuminemia and neutrophil cytoplasmic toxic changes were significantly associated with mortality. Cats exhibiting both conditions had a 120-fold increased risk of death compared to those without hypoalbuminemia and neutrophil cytoplasmic toxicity.

In this study, hypocalcemia did not demonstrate a significant difference between groups, nor did it emerge as a prognostic factor, contrary to previous studies [4,5]. Both total and ionized hypocalcemia have been reported as prognostic indicators in feline pancreatitis, with ionized calcium levels below 1 mmol/L being particularly associated with increased mortality [5,7]. However, a 2015 study [7] found no significant difference in ionized calcium levels between survivors and non-survivors on the first day of diagnosis. Instead, calcium levels ≤ 1 mmol/L measured on the final day—whether upon recovery or death (ranging from 2 to 11 days)—were predictive of mortality. A larger study in 2018 [4] included 152 cats but reported relatively few deaths (*n* = 35), and ionized calcium data were available for only 84 cats. Among non-survivors, hypocalcemia was observed in 4 out of 18 cases, while only 3 out of 66 survivors exhibited hypocalcemia, making the multivariable logistic regression analyses underpowered. These findings suggest that single-timepoint calcium measurements may not be sufficient predictors of mortality. Future studies should incorporate serial monitoring of ionized calcium levels to assess whether a progressive decline could serve as a valuable prognostic marker. Early identification of such changes may justify more intensive management strategies.

Previous studies have identified metabolic acidosis as a prognostic factor in acute pancreatitis in humans and dogs [11,12,13], though its role in cats remains unclear [5]. In this study, metabolic acidosis was more common in non-survivors; however, no significant difference in hyperlactatemia was observed between groups on the first day of hospitalization. Metabolic acidosis in acute pancreatitis may result from various mechanisms beyond lactic acidosis, including shock, renal failure, or the loss of bicarbonate-rich pancreatic secretions due to pancreatic duct disruption [11]. Moreover, lactate levels in cats may not follow a linear progression as seen in other species, potentially rising exponentially in cases of severe diseases. Consequently, cats with mild to moderate illnesses may exhibit only slight lactate elevations, yet a greater proportion of these individuals with modestly increased lactate levels may not survive compared to other species [49]. A 2018 review [49] suggested that serial lactate measurements and relative changes provide better prognostic accuracy than single-timepoint assessments. Future studies incorporating serial acid-base and lactate monitoring throughout hospitalization may offer further insight into the prognostic and therapeutic implications of these parameters.

This study had several limitations. First, data were retrospectively retrieved, and medical records were occasionally incomplete. This limitation may have influenced the findings, as additional treatments beyond the standard protocol, such as antimicrobial administration or blood transfusions, could have affected survival outcomes.

Second, the gold standard for diagnosing feline pancreatitis is histologic analysis of pancreatic biopsy specimens [10]. However, due to the challenges associated with obtaining biopsies, this method is not commonly used in clinical practice [50,51]. Instead, diagnosis in this study was based on a combination of clinical signs, an abnormal SNAP fPL test result, and abdominal ultrasonographic findings. It is important to note that the SNAP fPL test is a semiquantitative assay that categorizes results as either “normal” or “abnormal”. An “abnormal” result includes both equivocal (3.5–5.4 μg/L) and definitively positive (>5.4 μg/L) cases [52]. Without additional confirmatory testing, the proportion of false positives in this study could not be determined.

Third, this study focused exclusively on hospitalized cats with pancreatitis admitted to a critical care unit, which may have influenced the observed mortality rate of 55%. This rate is higher than the 9–41% mortality range reported in four previous studies [2,3,4,5] and likely reflects the increased disease severity in this study population. Admission decisions were made by the attending veterinarian based on the severity of pancreatitis, suggesting that the prognostic factors identified in this study may primarily apply to severe cases.

## 5. Conclusions

This study identified key clinical and clinicopathological variables that significantly differed between survivors and non-survivors in cats with pancreatitis. The primary objective was to determine prognostic factors in hospitalized cats with pancreatitis. Multivariate analysis revealed that jaundice, concurrent renal disease, hypoalbuminemia, increased BUN, and neutrophil cytoplasmic toxic changes upon admission were independent predictors of mortality. However, hyperlactatemia and metabolic acidosis upon admission were not associated with prognosis in hospitalized cats with pancreatitis.

## Figures and Tables

**Table 1:** Concurrent diseases in 142 hospitalized cats with pancreatitis.

Concurrent Disease	Survivors (*n* = 63)	Non-Survivors (*n* = 79)	All Cats (*n* = 142)	*p*-Value
	*n* (%)	*n* (%)	*n* (%)	
GI and GI-associated diseases	28/63 (44.4%)	39/79 (49.4%)	67/142 (47.2%)	0.5594
Renal disease	15/63 (23.8%)	31/79 (39.2%)	46/142 (32.4%)	0.0500
Endocrinopathy	20/63 (31.7%)	19/79 (24.1%)	39/142 (27.5%)	0.3074
Cardiological abnormality	7/63 (11.1%)	11/79 (13.9%)	18/142 (12.7%)	0.6167
Post-operative patient	12/63 (19.0%)	5/79 (6.3%)	17/142 (12.0%)	0.0204 *
FeLV positive	2/32 (3.2%)	12/32 (37.5%)	14/71 (19.7%)	0.098
FIV positive	3/33 (4.8%)	5/39 (12.8%)	8/65 (12.3%)	0.4752
Other conditions	16/63 (25.4%)	27/79 (34.2%)	43/142 (30.3%)	0.2579

* Significant difference between survival and non-survival groups (*p* < 0.05).

**Table 2:** Clinical signs and physical examination findings in 142 hospitalized cats with pancreatitis at presentation.

	Survivors (*n* = 63)	Non-Survivors (*n* = 79)	All Cats (*n* = 142)	*p*-Value
	*n* (%)	*n* (%)	*n* (%)	
Clinical signs				
Lethargy	52/63 (88.60%)	70/79 (88.61%)	122/142 (85.92%)	0.3017
Anorexia	49/63 (77.78%)	68/79 (86.08%)	117/142 (82.39%)	0.1971
Vomit	28/63 (44.44%)	36/79 (45.57%)	64/142 (45.07%)	0.8935
Diarrhea	10/63 (15.87%)	7/79 (8.86%)	17/142 (11.97%)	0.201
Weight loss	9/63 (14.28%)	7/79 (8.86%)	16/142 (11.27%)	0.3098
Physical examination findings				
Dehydration	47/63 (74.60%)	68/79 (86.06%)	115/142 (80.98%)	0.0835
Hypothermia	45/63 (71.43%)	65/79 (82.28%)	110/142 (77.46%)	0.1242
Abdominal pain	41/63 (65.08%)	59/79 (74.68%)	100/142 (70.42%)	0.2128
Icterus	7/63 (11.11%)	23/79 (29.11%)	30/142 (21.13%)	0.009 *
Fever	5/63 (7.94%)	2/79 (4.93%)	7/142 (4.93%)	0.1394

* Significant difference between survival and non-survival groups (*p* < 0.05).

**Table 3:** Complete blood count and blood chemistry in 142 hospitalized cats with pancreatitis.

Parameter		Survivors (*n* = 63)	Non-Survivors (*n* = 79)	All Cats (*n* = 142)	*p*-Value ^a^	*p*-Value ^b^
(Reference Range)						
Hematocrit (%)	Mean (SD)	29.3 (7.8)	26.1 (7.7)	27.5 (7.9)	0.01485 *	
(30–45%)	<30%; *n* (%)	35/63 (55.6%)	56/79 (70.9%)	91/142 (64.1%)		0.0585
Red blood cells (×10^6^/μL)	Mean (SD)	7.2 (2.0)	6.5 (2.2)	6.8 (2.1)	0.04252 *	
(5–10 × 10^6^/μL)						
Leukocytes (×10^3^/μL)	Median (IQR)	16.8 (11.3–27.3)	20.0 (13.3–27.1)	18.8 (12.5–27.2)	0.35447	
(5.5–19.5 × 10^3^/μL)	>19.5 × 10^3^/μL; *n* (%)	26/63 (41.3%)	40/79 (50.6%)	66/142 (46.5%)		0.2664
Neutrophils (×10^3^/μL)	Median (IQR)	13.3 (9.6–22.6)	16.0 (10.0–24.9)	15.4 (9.6–23.6)	0.38692	
(2.5–12.5 × 10^3^/μL)	>12.5 × 10^3^/μL; *n* (%)	32/63 (50.8%)	53/79 (67.1%)	85/142 (59.9%)		0.1084
	<2.5 × 10^3^/μL; *n* (%)	1/63 (1.6%)	2/79 (2.5%)	3/142 (2.1%)		0.1084
Lymphocytes (×10^3^/μL)	Median (IQR)	1.9 (0.9–2.7)	1.4 (0.7–3.3)	1.5 (0.9–2.9)	0.31342	
(1.5–7 × 10^3^/μL)						
Monocytes (×10^3^/μL)	Median (IQR)	0.4 (0.2–0.7)	0.4 (0.1–1.0)	0.4 (0.2–0.9)	0.72967	
(0–0.9 × 10^3^/μL)						
Eosinophils (×10^3^/μL)	Median (IQR)	0.3 (0.0–0.6)	0.2 (0.0–0.7)	0.2 (0.0–0.7)	0.26614	
(0–0.8 × 10^3^/μL)						
Platelets (10^3^/μL)	Median (IQR)	295 (201–436)	245 (108–403)	282 (140–416)	0.06704	
(200–500 × 10^3^/μL)						
BUN (mg/dL)	Median (IQR)	27 (22–32)	46 (33–63)	34 (20–78)	0.00281 *	
(19–34 mg/dL)	>34; *n* (%)	21/63 (33.3%)	47/79 (59.5%)	68/142 (47.9%)		0.0019 *
Creatinine (mg/dL)	Median (IQR)	1.4 (1.2–1.6)	1.9 (1.4–2.4)	1.6 (1.1–3.2)	0.04407 *	
(0.6–1.6 mg/dL)	>1.6; *n* (%)	24/63 (38.1%)	45/79 (57.0%)	69/142 (48.6%)		0.0254 *
ALT (U/L)	Median (IQR)	75 (59–112)	106 (83–137)	89 (47–171)	0.11666	
(6–70 U/L)	>70; *n* (%)	32/63 (50.8%)	50/73 (68.5%)	82/136 (60.3%)		0.0534
AST (U/L)	Median (IQR)	56 (28–172)	127 (54–375)	75 (54–213)	0.06916	
(7–38 U/L)	>38; *n* (%)	7/8 (87.5%)	11/11 (100%)	18/19 (94.7%)		0.4211
ALP (U/L)	Median (IQR)	40 (17–93)	86 (36–263)	56 (23–192)	0.07461	
(0–45 U/L)	>45; *n* (%)	11/23 (47.8%)	14/22 (63.6%)	25/45 (55.6%)		0.286
Albumin (g/dL)	Median (IQR)	3.0 (2.7–9-3.2)	2.5 (2.3–2.7)	2.8 (2.3–3.3)	0.00003 *	
(2.8–3.9 g/dL)	<2.8; *n* (%)	19/63 (30.2%)	51/79 (64.6%)	70/142 (49.3%)		0 *
Globulin (g/dL)	Median (IQR)	4.0 (3.7–4.2)	3.9 (3.5–4.2)	3.9 (3.3–4.5)	0.27712	
(2.6–5.1 g/dL)	>5.1; *n* (%)	4/63 (6.3%)	10/77 (12.9%)	14/140 (10.0%)		0.1928
Bilirubin (mg/dL)	Median (IQR)	2.4 (0.4–6.9)	4.3 (1.8–6.8)	3.8 (1.3–7.9)	0.44198	
(0–0.1 mg/dL)	>0.1; *n* (%)	14/16 (87.5%)	27/28 (96.4%)	41/44 (93.2%)		0.5433
Phosphorus (mg/dL)	Median (IQR)	6.4 (4.6–14.7)	7.5 (5.4–17)	7.5 (5.4–16.2)	0.47408	
(3.0–6.1 mg/dL)	>6.1; *n* (%)	6/9 (66.7%)	10/15 (66.7%)	16/24 (66.7%)		1

* Significant difference between survival and non-survival groups (*p*-value < 0.05). *p*-value ^a^ was a continuous value comparison between survival and non-survival groups. *p*-value ^b^ was a categorized variable comparison between survival and non-survival groups.

**Table 4:** Venous blood gas analysis and blood lactate parameters of 103 hospitalized cats with pancreatitis at presentation.

Parameter		Survivor Cats (*n* = 43)	Non-Survivor Cats (*n* = 60)	All Cats (*n* = 103)	*p*-Value ^a^	*p*-Value ^b^
(Reference Range)						
pH	Median (IQR)	7.34 (7.3–7.39)	7.30 (7.24–7.34)	7.33 (7.26–7.35)	0.01231 *	
7.22–7.38	<7.22; *n* (%)	5/43 (11.6%)	19/60 (31.7%)	24/103 (23.3%)		0.0177 *
CO_2_ (mmHg)	Mean (SD)	37.9 (6.5)	37.5 (9.1)	37.7 (8.1)	0.83054	
(41.0–50.8 mmHg)						
Ca^2+^ (mmol/L)	Median (IQR)	1.19 (1.15–1.25)	1.18 (1.11–1.22)	1.19 (1.15–1.127)	0.14012	
(1.13–1.38 mmol/L)	<1.13; *n* (%)	7/43 (16.3%)	22/60 (36.7%)	29/103 (28.2%)		0.0533
	<1.0; *n* (%)	2/43 (4.7%)	9/60 (15.0%)	11/103 (10.7%)		0.1154
K^+^ (mmol/L)	Median (IQR)	3.5 (3.1–3.8)	3.4 (3.18–3.8)	3.4 (3.0–3.8)	0.50982	
(3.0–4.8 mmol/L)	<3.0; *n* (%)	11/43 (25.6%)	13/60 (21.7%)	24/103 (23.3%)		0.643
	>4.8; *n* (%)	5/43 (11.6%)	11/60 (18.3%)	16/103 (15.5%)		0.3542
lactate	Median (IQR)	1.8 (1.5–2.2)	1.95 (1.4–2.5)	1.8 (1.5–2.6)	0.65876	
	>2.5 mmol/L	11/43 (25.6%)	23/60 (38.3%)	34/103 (33.0%)		0.1747
HCO_3_ (mmol/L)	Median (IQR)	20 (19.0–21.6)	18.8 (14.2–20.3)	19.2 (14.4–20.6)	0.02802 *	
(18.0–23.2 mmol/L)	<18.0; *n* (%)	10/43 (23.2%)	29/60 (48.3%)	39/103 (37.9%)		0.0097 *
glucose (mg%)	Median (IQR)	151 (120–226)	139 (117–164)	147 (118–230)	0.25837	
	<60; *n* (%)	3/43 (7.0%)	5/60 (8.3%)	8/103 (7.8%)		1.0000

* Significant difference between survival and non-survival groups (*p* < 0.05). *p*-value ^a^ was a continuous value comparison between survival and non-survival groups. *p*-value ^b^ was a categorized variable comparison between survival and non-survival groups.

**Table 5:** Association of multivariable outcomes in feline pancreatitis.

Variable	Regression Coefficient (β)	Standard Error	*p*-Value	Exp (β) OR	95% CI
Model 1: Clinical signs and physical examination findings					
Renal disease	1.05	0.40	0.009 *	2.86	0.26–1.84
Jaundice	1.49	0.50	0.003 *	4.44	0.51–2.48
Model 2: Clinicopathological data					
Neutrophil cytoplasmic toxic change	2.36	0.47	0.001 *	10.54	4.17–26.7
Azotemia (Increased BUN)	1.56	0.45	0.005 *	4.76	1.96–11.53
Hypoalbuminemia	1.16	0.48	0.016 *	3.91	1.70–9.01

* Significant difference between survival and non-survival groups (*p* < 0.05).

## Data Availability

The data presented in this study are included within the article. The raw data supporting the findings of this study are available from the corresponding author upon reasonable request.
